# Electrocardiographic and cardiometabolic risk markers of left ventricular diastolic dysfunction in physically active adults: CHIEF heart study

**DOI:** 10.3389/fcvm.2022.941912

**Published:** 2022-07-27

**Authors:** Pang-Yen Liu, Kun-Zhe Tsai, Wei-Chun Huang, Carl J. Lavie, Gen-Min Lin

**Affiliations:** ^1^Department of Medicine, Hualien Armed Forces General Hospital, Hualien City, Taiwan; ^2^Department of Medicine, Tri-Service General Hospital and National Defense Medical Center, Taipei, Taiwan; ^3^Department of Stomatology of Periodontology, Mackay Memorial Hospital, Taipei, Taiwan; ^4^College of Medicine, National Yang Ming Chiao Tung University, Taipei, Taiwan; ^5^Department of Critical Care Medicine, Kaohsiung Veterans General Hospital, Kaohsiung, Taiwan; ^6^John Ochsner Heart and Vascular Institute, Ochsner Clinical School, The University of Queensland School of Medicine, New Orleans, LA, United States

**Keywords:** cardiometabolic risk factors, electrocardiography, left ventricular diastolic dysfunction, physical fitness, young adult

## Abstract

**Aim:**

This study was aimed to investigate the association of cardiometabolic and ECG markers with left ventricular diastolic dysfunction (LVDD) in physically active Asian young adults, which has not been clarified in prior studies.

**Methods and results:**

A total of 2,019 men aged 18–43 years were included from the military in Taiwan. All the subjects underwent anthropometric, hemodynamic, and blood metabolic marker measurements. Physical fitness was investigated by time for a 3,000-m run. LVDD was defined by presence of either one of the three echocardiographic criteria: (1) mitral inflow E/A ratio < 0.8 with a peak E velocity of > 50 cm/s, (2) tissue Doppler lateral mitral annulus e′ <10 cm/s, and (3) E/e′ ratio > 14. Multiple logistic regressions with adjustments for age, physical fitness, and pulse rate were conducted to determine the association of cardiometabolic and ECG markers with LVDD. The prevalence of LVDD was estimated to be 4.16% (*N* = 84). Of the cardiometabolic markers, central obesity, defined as waist circumference ≥ 90 cm, was the only independent marker of LVDD [odds ratio (OR) and 95% confidence interval: 2.97 (1.63–5.41)]. There were no association for hypertension, prediabetes, and dyslipidemia. Of the ECG markers, left atrial enlargement and incomplete right bundle branch block/intraventricular conduction delay were the independent ECG markers of LVDD [OR: 2.98 (1.28–6.94) and 1.94 (1.09–3.47), respectively]. There was borderline association for Cornell-based left ventricular hypertrophy and inferior T wave inversion [OR: 1.94 (0.97–3.63) and 2.44 (0.98–6.08), respectively].

**Conclusion:**

In the physically active Asian young male adults, central obesity and some ECG markers for left heart abnormalities were useful to identify LVDD.

## Introduction

Left ventricular diastolic dysfunction (LVDD) is common in the early stage of heart failure (HF) (1). LVDD develops mostly due to stiff and reduced compliant left ventricle which impairs the relaxation or diastolic function and thus raises LV end-diastolic pressure as in rigorous exercise or stress (2). In addition, LVDD has been associated with greater risk of morbidity and mortality even in those with preserved LV ejection fraction (3, 4). The prevalence of LVDD in middle- or old-aged individuals is estimated to be 11–35% (4–6) when several cardiometabolic risk factors, e.g., obesity, diabetes, dyslipidemia, arterial hypertension, and coronary artery disease, commonly develop in midlife (7–9). In contrast, the Coronary Artery Risk Development in Young Adults (CARDIA) study revealed that the prevalence of LVDD in young adults with severe diastolic dysfunction was estimated only 1.1% and those with abnormal relaxation was estimated 9.3% (10).

The CARDIA study revealed that the LV diastolic filling of White and Black young adults is related to sex, age, body weight, systolic blood pressure (BP), heart rate, lung function, cardiac systolic function, and physical fitness but not related to electrocardiographic (ECG) LV mass index (10, 11). In addition, prolongation of corrected QT interval (QTc), on the basis of the Bazett's formula (12) representing relaxation of the LV and diastolic phase of electrical repolarization, has been identified as an independent ECG marker to predict the presence of LVDD in middle- and old-aged individuals (13, 14). Since the influences of physical fitness on cardiometabolic risk factors modifications, ECG changes and LVDD were rarely examined in Asian young adults, the aim of this study was to investigate the association of ECG and cardiometabolic markers with LVDD in a physically active military population in Taiwan.

## Methods

### Study population

The cross-sectional study included 2,688 military personnel aged 18–43 years from the cardiorespiratory fitness and health in eastern armed forces (CHIEF)-heart study in eastern Taiwan of ROC from 2018 to 2021 (15–17). Of them, 2,386 were men and 302 were women receiving daily physical training at their base. Each participant underwent annual health examinations for routine laboratory tests and self-reported their behavior, e.g., tobacco smoking and alcohol intake (active vs. former or never) in a questionnaire in the Hualien Armed Forces General Hospital. All the subjects participated in annual fitness exams for a 3,000-m run test to investigate their endurance capacity in the Hualien Military Physical Training and Testing Center (18, 19). After the exercise test, a 12-lead surface ECG and a transthoracic echocardiography were performed to assess each subject's cardiac structure and LV diastolic function before the end of the same year (20, 21). Participants were excluded if BP ≥ 140/90 mmHg (*N* = 346), body mass index (BMI) ≥ 35 kg/m^2^ (*N* = 32), or serum triglycerides ≥ 400 mg/dl (*N* =38), leaving a sample of 2,272 participants. Since there were merely 4 cases in 253 women fulfilling the criteria of LVDD in echocardiography, the group of women was further excluded from the analysis, leaving a sample of 2,019 men for the analysis.

### Physical examinations and blood biochemistry measurements

Each subject's anthropometric variables of body height, body weight, and waist circumference (WC) were measured in a standing position. BMI was calculated as the ratio of body weight (kg) to the square of body height (m^2^). The BP and pulse rate of each subject were measured once over the right arm in a sitting position at rest using an automatic BP monitor machine (FT201; Parama-Tech Co., Ltd., Fukuoka, Japan) on the basis of the oscillometric method. Routine blood tests, including serum uric acid (SUA), total cholesterol, serum triglycerides, high-density lipoprotein cholesterol (HDL-C), and fasting plasma glucose, were measured with an auto-analyzer (Olympus AU640; Kobe, Japan). Each subject's blood sample was obtained after an overnight 12-h fast in the same station.

### Cardiometabolic risk markers for LVDD

Metabolic syndrome was defined according to the latest criteria of International Diabetes Federation for Chinese (22) as having three or more clinical features: (1) fasting glucose ≥ 100 mg/dl or on anti-diabetic therapy, (2) HDL-C <40 mg/dl for men, (3) serum triglycerides ≥ 150 mg/dl or on lipid-lowering therapy, (4) systolic BP ≥ 130 mmHg and/or diastolic BP ≥ 85 mmHg or on antihypertensive therapy, and (5) central obesity: WC ≥ 90 cm for men. Hyperuricemia was defined as SUA ≥ 7 mg/dl for men (23).

### ECG and echocardiographic measurements

The ECG reports generated *via* the CARDIOVIT MS-2015 machine (Schiller AG, Baar, Switzerland) were reviewed and approved by a certificated cardiologist. Cornell ECG based-LVH in Asian young male adults was fulfilled if (R–aVL + S–V3) ≥ 18 mm (24), and Sokolow-Lyon ECG based-LVH was diagnosed if (S–V1 or S–V2 + R–V5 or R–V6) ≥ 35 mm (25). Sokolow-Lyon ECG based-RVH was defined if (R–V1 + S–V5 or S–V6) >10.5 mm (25), and Myers et al. ECG-based RVH was defined if (1) the R/S ratio of V1 > 1 or the R/S ratio of V5 or V6 < 1 or (2) R–V1 > 6 mm (25). ECG-based left atrial enlargement (LAE) was defined as a notched P wave in lead II ≥ 0.12 s or a notch of P wave ≥ 0.04 s (21). Inferior T wave inversion (TWI) was defined as one or more negative T wave axes in limb leads II, III, or aVF. First-degree atrioventricular block was diagnosed as a PR interval ≥ 200 ms. Complete and incomplete right bundle branch block (RBBB) should fulfill specific ECG patterns, e.g., Rsr of lead V1. The QRS duration of complete RBBB was ≥ 120 ms, and that of incomplete RBBB and intraventricular conduction delay (IVCD) ranged from 100 to 119 ms. A QTc interval ≥ 480 ms was defined as prolongation of QTc (26).

The transthoracic echocardiographic reports generated *via* the iE33 machine (Philips Medical Systems, Andover, MA, United States) were reviewed by a qualified cardiologist at the Hualien-Armed Forces General Hospital. According to the recommendations of the American Society of Echocardiography (27), LV mass was calculated using the corrected formula proposed by Devereux et al. (28): 0.8 × {1.04 × [(LV internal diameter (LVIDd) + posterior wall thickness + inter-ventricular septal thickness]^3^ – LVIDd^3^} + 0.6. The echocardiographic LVH index for body height (m^2.7^) was determined as ≥ 49 g/m^2.7^ for male adults (24). LVDD was defined as either one of the three criteria in echocardiography being fulfilled (29): (1) mitral inflow Doppler E/A ratio < 0.8 along with a peak E velocity of > 50 cm/s, (2) velocity of the lateral mitral annulus tissue Doppler, e′ < 10 cm/s, and (3) ratio of E/e′ > 14. Since our participants were physically active young men who might have a greater cardiac structure and function adaptations to exercise (20), the other two echocardiographic criteria for LVDD (29), (4) tricuspid regurgitation jet velocity > 2.8 m/s, and (5) left atrial volume index > 34 ml/m^2^, were still within the normal limits suggestive for athletes (30, 31) and not appropriate as inclusion criteria for LVDD in our military participants.

### Statistical analysis

The clinical characteristics and cardiac features of the military men were presented as mean ± standard deviation (SD) for continuous variables and numbers (%) for categorical variables. Univariate linear regression was conducted to determine the correlation of each cardiometabolic risk marker and ECG marker with LVDD. Multiple linear regression was conducted to determine the independent cardiometabolic risk biomarkers and abnormal ECG surrogates of LVDD. In addition, multivariable logistic regression was carried out to determine the odds ratio (ORs) of all cardiometabolic risk factors and all ECG markers separately, with adjustments for smoking, alcohol intake status, age, pulse rate, and physical fitness. A receiver operating characteristic (ROC) curve for each significant predictor of LVDD was used to determine their cut-off point, positive predictive value (PPV), and negative predictive value (NPV). A value of *P* < 0.05 was regarded significant. All the analyses were performed using SPSS version 25.0 for Windows (IBM Corp., Armonk, NY, United States). This study was carried out in compliance with the Declaration of Helsinki principles, and was approved by the Institutional Review Board of the Mennonite Christian Hospital (No. 16-05-008) in Hualien, Taiwan. Written informed consent was obtained from all the participants.

## Results

### Clinical characteristics and cardiovascular features

The clinical characteristics and CV features of the study population are shown in Table 1. The average age of the physically active men was 27.5 years. The mean pulse rate was 67.2 beats per min. In the study population, the prevalence of metabolic syndrome was ~12.6%. In total, there were 892 (44.2%) active smokers, 854 (42.3%) active alcohol consumers; 84 of the participants (4.16%) fulfilled the criteria for LVDD. In general, those with LVDD were older and had greater prevalence of cardiometabolic risk factors.

**Table 1:** Clinical characteristics of the military men.

	**Total population** **(*****N*** = **2,019)**	**Without LVDD (*****N*** = **1,935)**	**With LVDD (*****N*** = **84)**	***p*** **-value**
Age, years	27.53 ± 5.93	27.38 ± 5.86	31.78 ± 6.27	<0.001
Smoking, active (%)	892 (44.18)	850 (43.7)	39 (57.4)	0.02
Alcohol intake, active (%)	854 (42.30)	815 (41.9)	35 (51.5)	0.11
3,000-m running time, seconds	873.15 ± 93.58	871.39 ± 91.20	922.19 ± 139.44	<0.001
Body mass index, kg/m^2^	24.88 ± 3.72	24.74 ± 3.60	28.57 ± 4.86	<0.001
Waist circumference, cm	83.56 ± 9.63	83.21 ± 9.39	93.35 ± 10.88	<0.001
Pulse rate, beats per minute	67.24 ± 11.10	66.98 ± 10.92	74.04 ± 12.78	<0.001
Systolic blood pressure, mmHg	118.60 ± 13.14	118.23 ± 12.63	128.84 ± 21.33	<0.001
Diastolic blood pressure, mmHg	70.12 ± 10.28	69.78 ± 9.78	79.47 ± 17.18	<0.001
Total cholesterol, mg/dL	174.22 ± 34.09	173.84 ± 33.67	186.40 ± 43.75	0.003
High-density lipoprotein, mg/dL	49.36 ± 10.44	49.50 ± 10.44	46.09 ± 9.87	0.008
Serum triglycerides, mg/dL	108.29 ± 82.20	106.79 ± 80.32	149.85 ± 117.23	<0.001
Fasting plasma glucose, mg/dL	93.58 ± 11.61	93.34 ± 9.83	100.29 ± 34.70	<0.001
Serum uric acid, mg/dL	6.74 ± 1.35	6.72 ± 1.34	7.38 ± 1.42	<0.001
Metabolic syndrome (%)	255 (12.63)	223 (11.5)	24 (35.3)	<0.001
LVM, gram	148.34 ± 32.01	147.00 ± 29.95	178.79 ± 39.35	<0.001
**LV diastolic parameters**				
E velocity, cm/s	86.50 ± 24.30	86.82 ± 14.68	76.60 ± 106.63	0.001
A velocity, cm/s	50.33 ± 15.38	49.72 ± 10.22	67.39 ± 61.40	<0.001
e^′^ velocity, cm/s	17.57 ± 8.39	17.85 ± 8.37	9.30 ± 2.64	<0.001
a^′^ velocity, cm/s	8.78 ± 3.64	8.75 ± 3.64	9.55 ± 3.15	0.08
**LVDD**				
E/A <0.8	16 (0.79)	0 (0.0)	16 (19.0)	<0.001
e^′^ <10 cm/s	64 (3.17)	0 (0.0)	64 (76.2)	<0.001
E/e^′^>14	4 (0.20)	0 (0.0)	4 (4.8)	<0.001

*ECG, electrocardiography; LV, left ventricle; LVDD, left ventricular diastolic dysfunction; LVM, left ventricular mass*.

*LVDD was defined as either one of the three echocardiographic criteria fulfilled: (1) the mitral inflow E/A ratio <0.8; (2) tissue Doppler at the lateral mitral annulus e′ velocity <10 cm/s; and (3) the E/e′ ratio >14*.

### Correlations of cardiometabolic and ECG markers with LV diastolic function markers of LV in young men

The univariate and multivariable linear regression results of cardiometabolic and ECG markers with LV diastolic function markers are shown in Table 2. In the univariate analyses, all of the cardiometabolic markers were significantly correlated with E/A ratio, e′ velocity, and E/e′ ratio. In the multiple linear regression models for E/A ratio and E/e′ ratio, WC was the only independent cardiometabolic marker of E/A and E/e′ {β: −0.3 [95% confidence interval (CI): −0.41, −0.19] and 0.015 (95% CI: 0.005, 0.025), respectively}. With regard to e′, diastolic BP and WC were the independent predictors of e′ [β: −0.069 (95% CI: −0.116, −0.021) and −0.093 (95% CI: −0.138, −0.047), respectively].

**Table 2:** Correlations of cardiometabolic and electrocardiographic markers with left ventricular diastolic parameters in physically active young male adults.

	**E/A**					**e′**					**E/e′**				
	**Univariate**			**Multivariable**		**Univariate**			**Multivariable**		**Univariate**			**Multivariable**	
	***R***	β **(95% CI)**	***p***	β **(95% CI)**	***p***	***R***	β **(95% CI)**	***p***	β **(95% CI)**	***p***	***R***	β **(95% CI)**	***p***	β **(95% CI)**	***p***
**Cardiometabolic markers**															
Systolic BP	0.15	−0.009 (−0.012, −0.007)	<0.001	−0.001 (−0.004, 0.002)	0.61	0.14	−0.092 (−0.120, −0.064)	<0.001	−0.007 (−0.044, 0.030)	0.72	0.15	0.020 (0.014, 0.026)	<0.001	0.007 (−0.001, 0.016)	0.07
Diastolic BP	0.20	−0.015 (−0.019, −0.012)	<0.001	−0.004 (−0.008, 0.000)	0.06	0.19	−0.152 (−0.187, −0.117)	<0.001	−0.069 (−0.116, −0.021)	0.004	0.15	0.027 (0.019, 0.034)	<0.001	0.010 (0.000, 0.020)	0.061
Total cholesterol	0.13	−0.003 (−0.004, −0.002)	<0.001	−0.001 (−0.002, 0.000)	0.27	0.12	−0.030 (−0.040, −0.019)	<0.001	−0.010 (−0.022, 0.002)	0.09	0.07	0.004 (0.001, 0.006)	0.002	−2.337*10^−5^ (−0.003, 0.003)	0.98
HDL-C	0.08	0.006 (0.003, 0.009)	<0.001	0.001 (−0.002, 0.005)	0.44	0.08	0.063 (0.028, 0.098)	<0.001	0.027 (−0.012, 0.066)	0.17	0.09	−0.015 (−0.022, −0.007)	<0.001	−0.005 (−0.014, 0.003)	0.23
Triglycerides	0.13	−0.001 (−0.002, −0.001)	<0.001	4.574*10^−5^ (0.000, 0.001)	0.84	0.11	−0.011 (−0.015, −0.007)	<0.001	0.001 (−0.004, 0.007)	0.60	0.11	0.002 (0.001, 0.003)	<0.001	0.001 (−0.001, 0.002)	0.36
Fasting glucose	0.10	−0.007 (−0.010, −0.004)	<0.001	0.000 (−0.003, 0.003)	0.86	0.09	−0.062 (−0.094, −0.031)	<0.001	−0.004 (−0.036, 0.028)	0.78	0.08	0.012 (0.005, 0.019)	0.001	0.002 (−0.005, 0.009)	0.51
Serum uric acid	0.09	−0.057 (−0.082, −0.031)	<0.001	−0.020 (−0.045, 0.006)	0.12	0.08	−0.496 (−0.766, −0.226)	<0.001	−0.061 (−0.345, 0.222)	0.67	0.09	0.117 (0.059, 0.175)	<0.001	0.035 (−0.027, 0.098)	0.26
WC	0.21	−0.018 (−0.021, −0.014)	<0.001	−0.006 (−0.010, −0.002)	0.003	0.21	−0.182 (−0.220, −0.145)	<0.001	−0.093 (−0.138, −0.047)	<0.001	0.17	0.032 (0.024, 0.040)	<0.001	0.015 (0.005, 0.025)	0.004
**Electrocardiographic markers**															
P duration (ms)	0.07	−0.004 (−0.006, −0.002)	<0.001	−0.002 (−0.004, 0.001)	0.17	0.06	−0.032 (−0.056, −0.009)	0.007	−0.009 (−0.035, 0.017)	0.49	0.01	0.001 (−0.004, 0.006)	0.72	−0.004 (−0.009, 0.002)	0.19
PR interval (ms)	0.03	−0.001 (−0.003, 0.000)	0.15	0.001 (−0.001, 0.003)	0.20	0.06	−0.028 (−0.047, −0.008)	0.005	−0.008 (−0.030, 0.013)	0.44	0.05	0.004 (0.000, 0.009)	0.04	0.004 (−0.001, 0.008)	0.12
QRS duration (ms)	0.01	0.001 (−0.003, 0.004)	0.58	−0.002 (−0.006, 0.001)	0.17	0.02	−0.013 (−0.049, 0.024)	0.49	−0.034 (−0.070, 0.002)	0.067	0.02	0.004 (−0.004, 0.012)	0.36	0.006 (−0.002, 0.014)	0.12
QTc interval (ms)	0.16	−0.005 (−0.007, −0.004)	<0.001	0.001 (0.000, 0.003)	0.12	0.05	−0.017 (−0.033, −0.002)	0.02	0.022 (0.004, 0.040)	0.01	0.05	0.004 (0.000, 0.007)	0.02	−0.001 (−0.005, 0.003)	0.78
QRS axis (degree)	0.13	0.003 (0.002, 0.005)	<0.001	0.002 (0.001, 0.003)	0.001	0.11	0.031 (0.019, 0.043)	<0.001	0.019 (0.007, 0.031)	0.002	0.05	−0.003 (−0.005, 0.000)	0.03	−0.001 (−0.004, 0.002)	0.51

*Multiple regression analysis was used to determine the association of left ventricular diastolic parameters with age, 3,000-m running time, pulse rate, cigarette smoking, alcohol intake status and cardiometabolic markers (systolic BP, diastolic BP, total cholesterol, HDL-C, serum triglycerides, fasting glucose, serum uric acid, waist circumference). Another multiple regression analysis was used to determine the association of left ventricular diastolic parameters with age, 3,000-m running time, pulse rate, cigarette smoking and alcohol intake status and electrocardiographic markers*.

*BP, blood pressure; HDL-C, high-density lipoprotein cholesterol; WC, waist circumference*.

In the univariate analyses for the ECG markers, P duration, QTc interval, and QRS axis were significantly correlated with E/A ratio and e′ velocity. PR interval, rather than P duration, was significantly correlated with E/e′ ratio. In the multiple linear regression models for E/A ratio, QRS axis was the only independent ECG marker of E/A [β: 0.002 (95% CI: 0.001, 0.003)]. For e′, QTc interval and QRS axis were the independent ECG markers of e′ [β: −0.095 (95% CI: −0.17, −0.02), 0.072 (95% CI: 0.01, 0.135), and 0.022 (95% CI: 0.004, 0.04), respectively]. With regard to E/e′ ratio, there were no independent ECG makers of E/e′.

### Cardiometabolic markers for LVDD in young men

Table 3 shows the results of multiple logistic regression analysis for cardiometabolic markers of LVDD in young adults. Abdominal obesity was the only independent cardiometabolic risk marker of LVDD in model 1 [OR: 3.14 (95% CI: 1.73–5.69)]. An additional adjustment for physical fitness in model 2 did not alter the main results in model 1 [OR: 2.97 (95% CI: 1.63–5.41)]. There were no associations for hyperuricemia, prediabetes, hypertension, and dyslipidemia in models 1 and 2.

**Table 3:** Associations between cardiometabolic risk factors and echocardiographic left ventricular diastolic dysfunction in young men.

	**LVDD**			
	**Model 1**		**Model 2**	
**Cardiometabolic risk factors**	**OR (95% CI)**	***p*** **-value**	**OR (95% CI)**	***p*** **-value**
BP ≥ 130/85 mmHg	1.32 (0.73–2.38)	0.36	1.33 (0.73–2.40)	0.35
Total cholesterol ≥ 200 mg/dl	0.98 (0.54–1.78)	0.94	0.99 (0.54–1.80)	0.97
HDL-C <40 mg/dl	1.08 (0.53–2.18)	0.84	1.04 (0.51–2.12)	0.91
Serum triglycerides ≥ 150 mg/dl	1.01 (0.49–2.09)	0.98	1.03 (0.50–2.14)	0.92
Fasting glucose ≥ 100 mg/dl	1.67 (0.89–3.11)	0.10	1.69 (0.91–3.14)	0.10
Serum uric acid ≥ 7.0 mg/dl	1.47 (0.87–2.50)	0.15	1.46 (0.86–2.49)	0.16
Waist circumference ≥ 90 mg/dl	3.14 (1.73–5.69)	<0.001	2.97 (1.63–5.41)	<0.001
Metabolic syndrome	0.86 (0.32–2.28)	0.76	0.83 (0.31–2.22)	0.71

*Multiple logistic regressions were used to determine the association of cardiometabolic risk factors with LVDD*.

*Model 1 adjusted for age, pulse rate, BP ≥ 130/85 mmHg, total cholesterol ≥ 200 mg/dl, HDL-C < 40 mg/dl, serum triglycerides ≥ 150 mg/dl, fasting glucose ≥ 100 mg/dl, serum uric acid ≥ 7.0 mg/dl, waist circumference ≥ 90 mg/dl, metabolic syndrome, tobacco smoking and alcohol intake*.

*Model 2 adjusted for the covariates in model 1 and 3,000-m running time*.

*BP, blood pressure; HDL-C, high-density lipoprotein cholesterol*.

### ECG markers for LVDD in young men

The multiple logistic regression analysis results for the ECG markers of LVDD in men are shown in Table 4. In model 2, ECG-based LAE, incomplete right bundle branch block, and QTc interval prolongation were the independent ECG markers of LVDD [OR: 1.94 (95% CI: 1.09–3.47), 2.98 (95% CI: 1.28–6.94), and 8.87 (95% CI: 1.62–48.6), respectively]. In addition, the association for Cornell ECG-based LVH and inferior TWI was borderline significant [OR: 1.88 (95% CI: 0.97–3.63) and 2.44 (95% CI: 0.98–6.08); *p*-values = 0.061 and 0.056, respectively]. The additional adjustment for physical fitness in model 2 yielded similar results compared to model 1.

**Table 4:** Associations between electrocardiographic risk factors and echocardiographic left ventricular diastolic dysfunction in young men.

	**LVDD**			
	**Model 1**		**Model 2**	
	**OR (95% CI)**	***p*** **-value**	**OR (95% CI)**	***p*** **-value**
Sokolow-Lyon based LVH	0.94 (0.55–1.60)	0.81	0.96 (0.56–1.63)	0.86
Cornell based LVH	1.88 (0.98–3.63)	0.059	1.88 (0.97–3.63)	0.061
Cornell based RVH	1.30 (0.67–2.54)	0.44	1.29 (0.66–2.51)	0.46
Myers et al. based RVH	1.18 (0.51–2.72)	0.70	1.15 (0.50–2.68)	0.74
Sinus bradycardia	0.90 (0.35–2.30)	0.82	0.92 (0.36–2.35)	0.85
Ectopic P rhythm	1.10 (0.26–4.70)	0.89	1.11 (0.26–4.74)	0.88
Left atrial enlargement	2.02 (1.13–3.59)	0.01	1.94 (1.09–3.47)	0.02
First degree atrioventricular block	1.01 (0.30–3.47)	0.98	1.07 (0.31–3.68)	0.91
Left axis deviation	0.00 (0.00–0.00)	0.99	0.00 (0.00–0.00)	0.99
Right axis deviation	0.62 (0.22–1.74)	0.36	0.64 (0.23–1.79)	0.39
Complete RBBB	1.38 (0.39–4.82)	0.61	1.38 (0.40–4.18)	0.61
Incomplete RBBB or IVCD	3.02 (1.30–6.99)	0.01	2.98 (1.28–6.94)	0.01
QTc prolongation >480 ms	10.31 (1.96–54.28)	0.006	8.87 (1.62–48.63)	0.01
Inferior T wave inversion	2.47 (0.99–6.17)	0.053	2.44 (0.98–6.08)	0.056

*Multiple logistic regressions were used to determine the association of electrocardiographic risk factors with left ventricular diastolic dysfunction*.

*Model 1 adjusted for age, pulse rate, smoking and alcohol intake status*.

*Model 2 adjusted for the covariates in model 1 and 3,000-m running time*.

*IVCD, intraventricular ventricular conduction delay; LVH, left ventricular hypertrophy; RBBB, right bundle branch block; RVH, right ventricular hypertrophy*.

### Cut-off points, PPV, and NPV for the predictors of LVDD by ROC analysis

Table 5 shows the results of cut-off values, PPV, and NPV for the independent predictors of LVDD in the multiple linear regression analysis by ROC analysis. The predictors included age, pulse rate, physical fitness (time for the 3,000-m run test), diastolic BP, WC, QTc interval and QRS axis, and area under curves of ROC for the predictors ranged from 0.62 to 0.76. Using the cut-off value for each predictor for LVDD, the NPV of each predictor was >95%, and the PPV of each predictor was <10%, in which the greatest PPV was observed in WC.

**Table 5:** Cut-off values, positive predictive values and negative predictive values for the predictors of left ventricular diastolic dysfunction by the receiver operating characteristic curve analysis.

	**AUC**	**95% CI**	***p*** **-value**	**Cut-off value**	**PPV**	**NPV**
Age, yrs	0.70	0.64–0.77	<0.001	25.5	5.2%	98.8%
Pulse rate, beats/min	0.66	0.60–0.72	<0.001	65.5	4.9%	98.4%
Time for a 3,000-m run, sec	0.62	0.55–0.69	0.001	869.5	2.2%	95.3%
Diastolic BP, mmHg	0.67	0.60–0.75	<0.001	74.5	6.5%	97.9%
Waist circumference, cm	0.76	0.70–0.82	<0.001	90.8	9.3%	98.3%
QTc interval, mm	0.67	0.60–0.74	<0.001	401.5	6.2%	98.0%
QRS axis, degree	0.68	0.61–0.75	<0.001	30.5	8.7%	97.4%

*AUC, area under curve, BP, blood pressure; CI, confidence interval; NPV, negative predictive value; PPV, positive predictive value*.

## Discussion

The principal findings of this study were that in the physically active men, diastolic BP and WC were the independent cardiometabolic markers, and that the QTc interval and QRS axis were the independent ECG markers correlated with the echocardiographic LV diastolic parameters. In addition, abdominal obesity was the only independent cardiometabolic risk factor, and ECG-based LAE, incomplete RBBB/IVCD, and QTc prolongation were the independent ECG risk factors of LVDD. There might be a suggestion that Cornell ECG-based LVH and inferior TWI were likely the ECG risk factors of LVDD in young men.

Several prior studies have revealed that diastolic BP was stronger than systolic BP in predicting the occurrence of CV disease (CVD) in young adults (32–34). In the CARDIA study, the association of CVD with diastolic BP being stronger than with systolic BP was only presented in White young adults and not in Black ones, indicating a racial difference (35). However, there were rare studies for Asian young adults. In a population study for Korean young adults, the risks of CVD between stage I isolated diastolic and systolic hypertension were similar (36). This study further emphasized that diastolic BP rather than systolic BP was independently and negatively correlated with e′, an LV diastolic parameter that was regarded as an early sign of LVDD and HF in young adults. In addition, central obesity has been well-known as a more potent risk marker than BMI in predicting LVDD (37). In this study, there was also a suggestion of an association between LVDD and inferior TWI, possibly because of leftward and horizontal displacement of the heart base in central obesity (19).

For the ECG markers, IVCD, Cornell-based LVH, and LAE might represent the status of great LV mass and pressure overload in the left heart (20). Incomplete RBBB and IVCD might reflect an increase in pulmonary artery pressure radiating from the left heart in the absence of RVH (38). As observed in prior studies on middle- and old aged individuals, prolongation of QTc, standing for abnormal LV relaxation, was also an independent risk factor of LVDD in physically active young adults in this study. In contrast to the CARDIA study (10, 11), we found that some ECG markers that were highly related to left heart abnormalities were useful to identify LVDD in Asian young adults. As the echocardiographic criteria (29) by tissue Doppler for LVDD were developed after 2016, there were no available data for tissue Doppler-defined LVDD in the CARDIA study. In this study, there were about three-fourths of LVDD cases diagnosed on the basis of the tissue Doppler criteria, indicating that tissue Doppler on the mitral annulus for e′ velocity may be a more sensitive tool for LVDD in young adults.

### Study strengths and limitations

The major strength of this study was that the subjects were included from the military in which the training programs and living circumferences were similar, possibly eliminating unrecognized confounders. In contrast, there were some limitations in this study. First, the study included only men and the results could not be applied to women. Second, since this study had a cross-sectional design, temporal associations for the changes in LV diastolic function could not be assessed. Third, some subjects of LVDD might not be taken into account, since the criteria of greater tricuspid regurgitation velocity and left atrial volume index for LVDD (39, 40) were not regarded as inclusion criteria in this study. For instance, there were 384 subjects (19%) with a tricuspid regurgitation velocity > 2.8 m/s, which was possibly due to the effect of athletes' heart. Finally, since this study included physically active military men only, the generalizability might not be applicable to the general population of young adults.

## Conclusion

Our study suggested that in physically active young men, central obesity and some ECG markers for left heart abnormalities were useful to identify LVDD. Whether some of the findings for the cardiometabolic and ECG markers were specific to Asian young adults should be verified in other Asian populations.

## Data availability statement

The raw data supporting the conclusions of this article will be made available by the authors, without undue reservation.

## Ethics statement

The studies involving human participants were reviewed and approved by the Institutional Review Board (IRB) of the Mennonite Christian Hospital (No. 16-05-008) in Hualien of Taiwan approved access to the data for the CHIEF study, and written informed consent was obtained from all participants. The patients/participants provided their written informed consent to participate in this study.

## Author contributions

P-YL and G-ML wrote the article. K-ZT conducted the statistical analyses. W-CH interpreted the data. CL raised critical comments and edited the manuscript. G-ML was the principal investigator for the study. All authors contributed to the article and approved the submitted version.

## Funding

This study was supported by the Medical Affairs Bureau Ministry of National Defense (MND-MAB-D-11115) and Hualien Armed Forces General Hospital (HAFGH-D-111003), where the main place was involved in the study design, data collection, analyses, and writing of this research.

## Conflict of interest

The authors declare that the research was conducted in the absence of any commercial or financial relationships that could be construed as a potential conflict of interest.

## Publisher's note

All claims expressed in this article are solely those of the authors and do not necessarily represent those of their affiliated organizations, or those of the publisher, the editors and the reviewers. Any product that may be evaluated in this article, or claim that may be made by its manufacturer, is not guaranteed or endorsed by the publisher.
