# Supplementary material for: Electrocardiographic and cardiometabolic risk markers of left ventricular diastolic dysfunction in physically active adults: CHIEF heart study
Source: Front Cardiovasc Med. 2022 Jul 27;9:941912. doi: 10.3389/fcvm.2022.941912 (PMC9363619; doi:10.3389/fcvm.2022.941912)
Supplement: Supplementary file 1 [file Table_1.pdf]

Supplemental Table. Correlations between Cardiometabolic Risk Factors and LV Diastolic Parameters in Men

	E/A				e′				E/e′			
	Multiple Linear Regression for cardiometabolic markers		Multiple Linear Regression for ECG markers		Multiple Linear Regression for cardiometabolic markers		Multiple Linear Regression for ECG markers		Multiple Linear Regression for cardiometabolic markers		Multiple Linear Regression for ECG markers	
	β (95% CI)	p	β (95% CI)	p	β (95% CI)	p	β (95% CI)	p	β (95% CI)	p	β (95% CI)	p
Age	-0.033<br>(-0.039, -0.027)	<0.01	-0.038<br>(-0.044, -0.032)	<0.001	-0.661<br>(-0.860, -0.463)	<0.001	-0.821<br>(-1.009, -0.632)	<0.001	0.022<br>(0.008, 0.037)	0.003	0.038<br>(0.024, 0.052)	<0.001
Time for a run	$1.886*10^{-5}$<br>(0.000, 0.000)	0.91	$-5.047*10^{-5}$<br>(0.000, 0.000)	0.77	0.006<br>(-0.006, 0.017)	0.32	0.005<br>(-0.006, 0.017)	0.36	0.001<br>(0.000, 0.002)	0.12	0.001<br>(0.000, 0.002)	0.02
Pulse rate	-0.016<br>(-0.019, -0.013)	<0.001	-0.019<br>(-0.022, -0.015)	<0.001	-0.149<br>(-0.245, -0.053)	0.002	-0.203<br>(-0.314, -0.093)	<0.001	0.001<br>(-0.006, 0.008)	0.76	0.008<br>(-0.001, 0.016)	0.07
